# The clinical treatment and outcome of cerebellopontine angle medulloblastoma: a retrospective study of 15 cases

**DOI:** 10.1038/s41598-020-66585-7

**Published:** 2020-06-17

**Authors:** Tao Wu, Pei-ran Qu, Shun Zhang, Shi-wei Li, Jing Zhang, Bo Wang, Pinan Liu, Chun-de Li, Fu Zhao

**Affiliations:** 1 Department of Neural Reconstruction, Beijing Key Laboratory of Central Nervous System Injury, Beijing Neurosurgical Institute, Capital Medical University, Beijing, 100070 China; 2 Department of Neurosurgery, Beijing Tian Tan Hospital, Capital Medical University, Beijing, 100070 China; 3 Department of Neurosurgery, Beijing Children’s Hospital, Capital Medical University, Beijing, 100045 China

**Keywords:** CNS cancer, Cancer imaging

## Abstract

Medulloblastoma (MB) is the most common malignant pediatric brain tumor arising in the cerebellum or the 4^th^ ventricle. Cerebellopontine angle (CPA) MBs are extremely rare tumors, with few cases previously described. In this study, we sought to describe the clinical characteristics, molecular features and outcomes of CPA MB. We retrospectively reviewed a total of 968 patients who had a histopathological diagnosis of MB at the Beijing Neurosurgical Institute between 2002 and 2016. The demographic characteristics, clinical manifestations and radiological features were retrospectively analyzed. Molecular subgroup was evaluated by the expression profiling array or immunohistochemistry. Overall survival (OS) and progression-free survival (PFS) were calculated using Kaplan-Meier analysis. In this study, 15 patients (12 adults and 3 children) with a mean age at diagnosis of 25.1 years (range 4–45 years) were included. CPA MBs represented 1.5% of the total cases of MB (15/968). Two molecular subgroups were identified in CPA MBs: 5 WNT-MBs (33%) and 10 SHH-MBs (67%). CPA WNT-MBs had the extracerebellar growth with the involvement of brainstem (*P* = 0.002), whereas CPA SHH-MBs predominantly located within the cerebellar hemispheres (*P* = 0.004). The 5-year OS and PFS rates for CPA MB were 80.0% ± 10.3% and 66.7% ± 12.2%, respectively. Pediatric patients with CPA MBs had worse outcomes than adult patients (OS: *P* = 0.019, PFS: *P* = 0.078). In conclusion, CPA MB is extremely rare and consists of two subgroups. Adult patients with CPA MB had a good prognosis. Maximum safe surgical resection combined with adjuvant radiotherapy and chemotherapy can be an effective treatment strategy for this rare tumor.

## Introduction

Medulloblastoma (MB) represents nearly 33% of pediatric and 1% of adult brain tumors and is associated with high morbidity and mortality^1^. More than 80% of pediatric MBs develop at the cerebellar vermis or the apex of the fourth ventricle^2–5^, whereas nearly 54% of cases are paramedian or lateral in adults^6^. In recent years, MB has been stratified into at least four molecular subgroups (WNT, SHH, Group 3, and Group 4) with distinct underlying biology and outcomes^7,8^. Interestingly, several studies demonstrate that MBs of the different molecular subgroups show distinct localizations within the hindbrain: Group 3 and Group 4 tumors are primarily midline and occupy the fourth ventricle, while a cerebellar hemispheric location is characteristic of SHH tumors^2,6,9,10^.

MBs located laterally in the cerebellopontine angle (CPA) are extremely rare. To date, only 48 cases of CPA MB have been reported in the English literature^11–18^. CPA MBs can occur in both children and adults who usually present with symptoms of raised intracranial pressure and cerebellar dysfunction along with dysfunctions of cranial nerves^11^. The preoperative diagnosis for CPA MB is difficult because the clinical presentations and radiographic features are similar to those of other common CPA tumors, such as schwannomas and meningiomas. More importantly, gross total resection (GTR) for CPA MBs is challenging because it carries a highly significant risk of injuring the facial nerve and adjacent vital brain structures, although the maximal extent of surgical resection remains the standard of care for this malignant tumor^19,20^.

Our previous MR imaging data demonstrate that a small portion of adult WNT- and SHH-MBs occur along the CP/CPA^6^. In this study, we described a series of patients with CPA MB who underwent a suboccipital approach for tumor removal at Beijing Tian Tan Hospital between 2002 and 2016. We reported the clinicopathologic and radiological features, molecular characteristics, treatments, and outcomes to explore potential management strategies for this group of patients.

## Patients and Methods

### Patient population

This study was approved by the ethics committee of the Beijing Tian Tan Hospital, Capital Medical University (Approval Number: KY2018-020-01), and informed consent was obtained from all patients or families. We retrospectively reviewed a cohort of 968 patients with a final pathological diagnosis of MB at the Beijing Neurosurgical Institute between 2002 and 2016 that included 214 adult patients and 754 pediatric patients. Patients with both preoperative MRI data and surgical tissue available for molecular analysis were included. All tumor specimens were sterilely stored (frozen or formalin-fixed paraffin-embedded tissues) at the Beijing Neurosurgical Institute in accordance with the Ethics Review Board of the Beijing Tian Tan Hospital (study reference).

### Radiologic evaluation

All MRI examinations were performed with a 1.5 T magnetic resonance system (Signa EchoSpeed, Version 8.2.3 software; GE Healthcare, Milwaukee, US) with a standard head coil. MRI data including T1, T2, diffusion-weighted imaging (DWI) and T1 sequences with gadolinium enhancement were collected preoperatively. The enhancement pattern was defined as minimal or none if less than 5% of the tumor volume was enhanced, solid if enhancement was present in more than 90% of the tumor volume, and heterogeneous if only patchy areas were enhanced. Ill-defined or well-defined tumor margins were characterized if more than 50% of the margin fit the description, and “margin” referred to the tumor margin against the adjacent cerebellum or brainstem. The growth pattern localization of the tumor was defined as intracerebellar growth, extracerebellar growth or intracerebellar and extracerebellar growth. Other MRI assessments included the presence of cysts or cavities and edema in the adjacent cerebellum. A CT scan was used to detect areas of hemorrhage and mineralization.

### Pathological and molecular evaluations

Histological diagnoses according to the criteria of the 2016 WHO classification were confirmed by two neuropathologists. The tumors were classified by histology as classic (C), desmoplastic/nodular (DN), anaplastic (A), or large cell (LC) MB. Molecular classification was established by using the Agilent Whole Human Genome Oligo Microarray Kit, 4 × 44 K (Gene Expression Omnibus accession No. GPL6480; Agilent Technologies, Santa Clara, CA, USA) and immunohistochemistry with antibodies for CTNNB1 (WNT/wingless marker, 1:100; ab610154, BD Transduction Laboratories), SFRP1 (SHH marker, 1:2000; ab4193, Abcam), NPR3 (subgroup 3 marker, 1:200; ab37617, Abcam), and KCNA1 (subgroup 4 marker, 1:2000; ab32433, Abcam), as we described previously^21^.

### *TP53* mutation analysis

To detect mutations of the *TP53* gene in SHH-MB tumors, genomic DNA derived from formalin-fixed paraffin-embedded (FFPE) tumor samples was prepared with the Wizard^®^ Genomic DNA Purification Kit (A1120, Promega, US) according to the manufacturer’s protocols. *TP53* sequencing was performed on the entire coding sequence (exons 2 through 11) with primers and methodology as previously described^22^. The PCR profile was performed as follows: 95 °C for 2 min and 56 °C for 1 min for 35 cycles. The final extension was added at 72 °C for 10 min before storage at 4 °C. The PCR products were loaded onto a 1% agarose gel for electrophoresis. The reactions were run and analyzed by an automated Genetic Analyzer ABI 310 system (ABI, CA) according to the manufacturer’s instructions.

### Follow-up

All patients were followed via telephone interviews or at the outpatient department to acquire more information. All available follow-up data began from initial diagnosis until subsequent tumor progression, patient death, or the end of the last follow-up. Enhanced MRI was performed for postoperative evaluation or in cases of the appearance of new neurological deficits. Facial motor function was assessed using the House and Brackmann (HB) classification. The audiometric examination was evaluated using pure tone audiometry (PTA) and the auditory brainstem response (ABR).

### Statistical analysis

Patient demographics and patient medical characteristics were summarized using descriptive statistics for quantitative data (mean ± standard deviation for normally distributed data and median for unknown distributed data) and qualitative data (count and percentage). All statistical analyses were performed using statistics software (Statistical Package for the Social Sciences Statistics, Version 22.0; IBM, Armonk, New York). A two-tailed *P* value < 0.05 was considered statistically significant. Fisher’s exact test was used to compare the categorical variable data. Overall survival (OS) and progression-free survival (PFS) were calculated using the Kaplan-Meier method (estimated 5-year PFS and OS rates), and data are reported as the mean ± standard error (SE). The log-rank test was used for comparison. OS for all analyses was defined as the time from diagnosis until death. PFS was defined as the time from the date of surgical resection until the date of tumor progression confirmed by imaging.

## Results

### Demographics and clinical characteristics

Of 968 patients diagnosed with primary MB, 15 patients had MBs located in the CPA (Table 1). There were 3 children and 12 adults with a mean age of 25.7 ± 11.8 years at surgery (ranging from 4–45 years). The male to female ratio was 1.1:1.

**Table 1:** Clinical and pathological features of patients with cerebellopontine angle medulloblastoma.

No.	Sex	Age	Surgical resection	CN involved	Histology	Molecular subgroup	Adjuvant therapy	Survival	Follow-up (mo.)
1	M	21 yrs	NTR	/	CMB	WNT	RT + CT	Alive	135
2	M	30 yrs	GTR	5^th^,7^th^,8^th^	CMB	WNT	RT + CT	Alive	120
3	F	16 yrs	NTR	7^th^,8^th^	CMB	WNT	RT + CT	Alive	74
4	M	19 yrs	GTR	/	CMB	WNT	RT + CT	Alive	64
5	M	19 yrs	NTR	/	CMB	WNT	RT + CT	Alive	40
6	M	45 yrs	NTR	/	CMB	SHH	RT + CT	Died	18
7	F	42 yrs	GTR	7^th^,8^th^, LCNs	CMB	SHH	RT + CT	Alive	98
8	F	34 yrs	NTR	5^th^,6^th^,7^th^,8^th^	CMB	SHH	RT + CT	Alive	91
9	M	24 yrs	GTR	5^th^,7^th^,8^th^	DNMB	SHH	RT + CT	Alive	71
10	M	29 yrs	GTR	/	DNMB	SHH	RT + CT	Alive	69
11	F	11 yrs	NTR	5^th^,6^th^,7^th^,8^th^	CMB	SHH	RT + CT	Died	33
12	F	19 yrs	GTR	/	CMB	SHH	RT + CT	Alive	54
13	F	38 yrs	NTR	/	DNMB	SHH	RT + CT	Alive	41
14	F	4 yrs	NTR	/	CMB	SHH	RT	Died	7
15	M	34 yrs	GTR	7^th^,8^th^	CMB	SHH	RT + CT	Alive	110

Abbreviations: CMB, classic medulloblastoma; CN, cranial nerve; CT, chemotherapy; DNMB, desmoplastic/nodular medulloblastoma; F, female; LCNs, lower cranial nerves; M, male; GTR, gross total resection; NTR, near-total resection; RT, radiotherapy.

Preoperatively, intracranial pressure (such as headache, vomiting and blurred vision) was found in all cases (100%), and cerebellar signs (ataxia, dizziness, and nystagmus) were found in 14 cases (93.3%). Diplopia was implicated in 4 cases (26.7%), and facial nerve and trigeminal nerve palsy was observed in 1 case (6.7%). The symptom duration ranged from 0.5 months to 10 months, with a median time of 2.7 months. The preoperative audiometric examinations, including PTA and the ABR, were abnormal in 4 cases (26.7%). No substantial difference in clinical features was observed between adult and childhood patients (Supplementary Table 1).

### Radiological features

**Figure 1:**
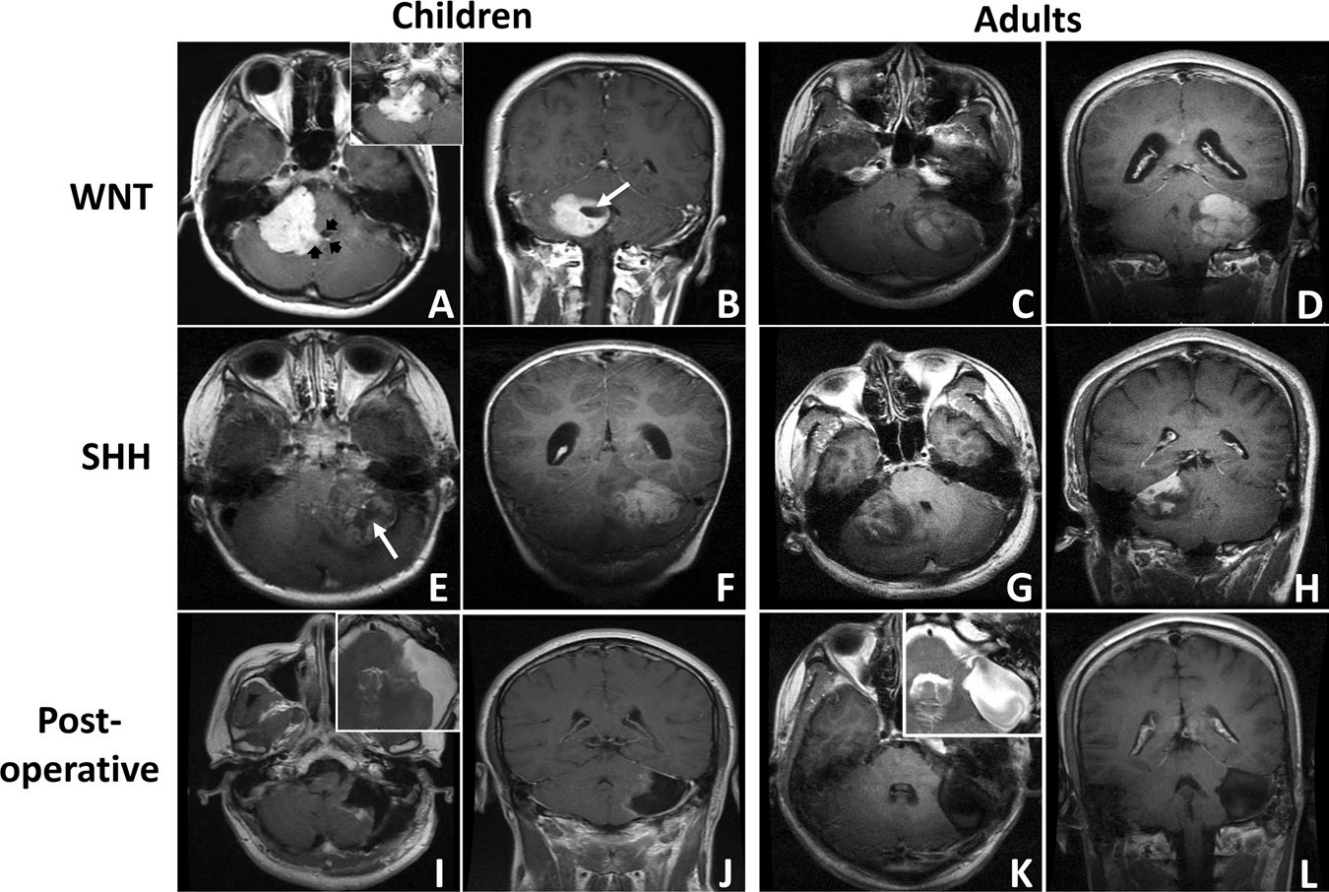
Exemplary magnetic resonance imaging (MRI) of cerebellopontine angle medulloblastoma (MB) (T1-weighted post-contrast axial and coronal MRIs). (**A**,**B**) A pediatric CPA WNT-MB with solid enhancement is shown (black arrow: the invasion of the 4^th^ ventricle is shown; white arrow: the focal cyst is shown; inset: prepontine cistern involvement). (**C**,**D**) An adult CPA WNT-MB with solid enhancement is shown (white arrow: the focal cyst is shown). (**E**,**F**) A pediatric CPA SHH-MB with heterogeneous enhancement is shown. (**G**,**H**) An adult CPA SHH-MB with heterogeneous enhancement is shown. (**I**,**J**) Postoperative images of a pediatric CPA SHH-MB are shown (inset: T2-weighted MRI; preoperative images: **E**,**F**). Damage to the left cerebellar hemisphere is shown. (**K**,**L**) Postoperative images of an adult CPA WNT-MB are shown (inset: T2-weighted MRI; preoperative images: **C**,**D**). Compression of the left cerebellar hemisphere without erosion is shown.

**Figure 2:**
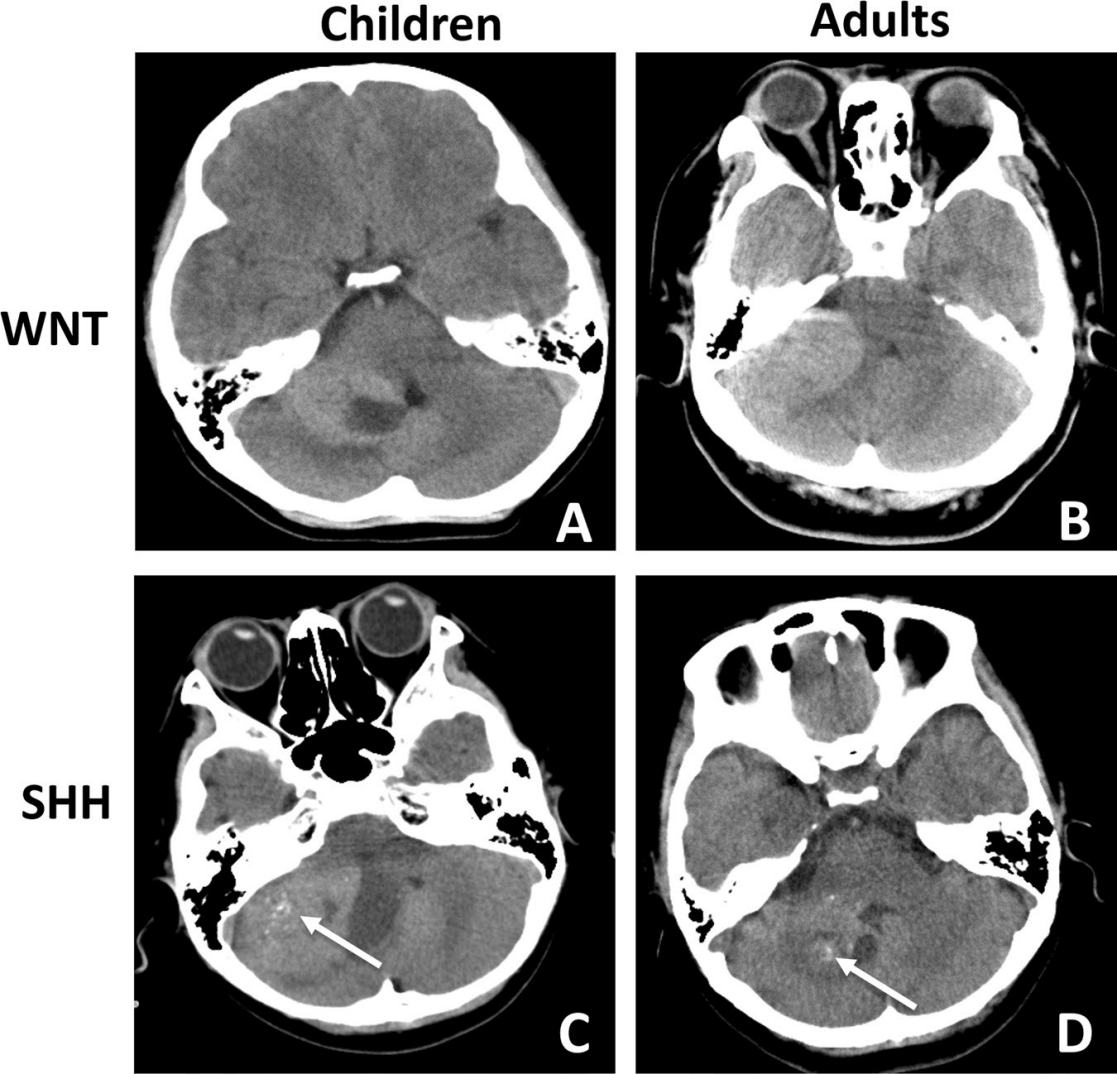
Exemplary CT scan (axial) of cerebellopontine angle medulloblastoma (MB). (**A**,**B**) Pediatric and adult CPA WNT-MBs are shown. (**C**,**D**) Pediatric and adult CPA SHH-MBs with mineralization (white arrow) are shown. No obvious bone erosion or dilation of the IAC is found in any of the cases.

The MRI study showed that 9 tumors (60.0%) were located on the left, and 6 tumors (40.0%) were located on the right (Fig. 1A–H). The mean tumor diameter was 4.4 ± 0.87 cm (range 3.5–5.7 cm). A well-defined margin was observed in 8 cases (53.3%), and peritumoral edema was seen in 7 cases (46.7%). None or small focal cysts (<1 cm) were present in 6 cases (40.0%), whereas cysts larger than 1 cm were present in 9 cases (60.0%; Fig. 1B,E, white arrow). After contrast administration, solid enhancement was seen in 8 cases (53.3%; Fig. 1A–D), while heterogeneous or minimal enhancement was seen in 7 cases (46.7%; Fig. 1E–H). The floor of the 4^th^ ventricle was involved in 3 cases (20.0%), while the prepontine cistern was involved in 1 case (6.7%; Fig. 1A, black arrow). Spinal metastasis was found in 1 case (6.7%). CT scans showed that lesions with high or heterogeneous density located in the CPA, coming into contact with the posterior edge of the petrous bone (Fig. 2). Hemorrhage or mineralization was identified in 5 cases (33.3%; Fig. 2C,D, white arrow). The IAC was not dilated in all cases. No substantial difference in radiological features was observed between adult and childhood patients (Supplementary Table 1).

### Surgical resection

Preoperative cerebrospinal fluid (CSF) diversion (ventriculoperitoneal shunting) was performed in 3 patients (20.0%). All patients underwent tumor resection through a standard retrosigmoid craniectomy with systematic intraoperative monitoring of the facial nerve using a four-channel NIM TM 2.0 (Medtronic). The ABR combined with the cochlear nerve action potential (CNAP) was used in cases requiring hearing preservation surgery. During surgery, adherence of the tumor to the facial nerve and vestibulocochlear nerve was observed in 46.7% of cases (Table 1). Furthermore, the cerebellar hemisphere was invaded in 9 cases (60.0%), and the brainstem was involved in 6 cases (40.0%). The 4^th^ ventricle was invaded through the foramen of Luschka in 3 cases (20.0%; Fig. 1). GTR was performed in 7 patients (46.7%; Fig. 1), and near-total removal (<1.5 cm; NTR) was performed in 8 patients (53.3%). GTR was not possible in cases where adhesion to the brainstem was severe (2 cases) or when acute severe hemorrhage occurred (1 case). NTR was performed deliberately in 5 cases (including 2 pediatric patients) for anatomic preservation of the facial nerve and vestibulocochlear nerve.

Postoperatively, symptoms of intracranial pressure and cerebellar signs were improved in all cases (100%), and diplopia and hearing function were improved in 2 and 1 cases, respectively. Temporary facial nerve paralysis (HB Grade II) was observed in 2 cases and recovered in 6 months. Facial nerve dysfunction (HB grade IV) and unilateral hearing loss were observed in 1 case. No severe complications such as cerebrospinal fluid leakage or intracranial infection were observed. No delayed obstructive hydrocephalus developed during follow-up.

### Histology and molecular classification

Histopathology revealed classic features of MB (CMB) in 12 cases (80.0%), while 3 cases had a diagnosis of DNMB (20.0%; Table 1). Molecular classification analysis showed that 15 CPA MBs consisted of 5 WNT tumors (33.3%) and 10 SHH tumors (66.7%). No significant difference in histopathological subtype or molecular subgroup was observed between adult and childhood patients (Supplementary Table 1). A striking association between biologic subgroups and radiological features was observed. Based on the pre- and postoperative MRI results and intraoperative reports, we found that all WNT-MBs were derived from the brainstem *(P* = 0.004) and grew exclusively outside the cerebellum, whereas SHH-MBs resided in the cerebellar hemispheres (*P* = 0.002; Fig. 1; Table 2). Moreover, solid enhancement was observed in all WNT-MBs (6/6; 100.0%) and in 30% of SHH-MBs (3/10; *P* = 0.044; Fig. 1). No significant difference was observed between two molecular subgroups according to the other clinical and radiological features (Table 2).

**Table 2:** The comparison of clinical characteristics between cerebellopontine angle WNT- and SHH-medulloblastomas.

Characteristics	WNT (n = 5) No. of patients (%)	SHH (n = 10) No. of patients (%)	P*
Sex			
Female	1 (20)	6 (60)	0.282
Male	4 (80)	4 (40)	
Age at diagnosis			
<18	1 (20)	2 (20)	1.000
≥18	4 (80)	8 (80)	
Origin^#^			
Cerebellum	0 (0)	9 (90)	**0.002**
Brainstem	5 (100)	1 (10)	
Growth pattern			
ICB/ICB + ECB	1 (20)	10 (100)	**0.004**
Only ECB	4 (80)	0 (0)	
4^th^ ventricle floor involvement			
Yes	3 (60)	1 (10)	0.077
No	2 (40)	9 (90)	
CN involvement			
Yes	2 (40)	5 (50)	0.573
No	3 (60)	5 (50)	
Cystic			
Yes	3 (20)	6 (60)	1.000
No	2 (80)	4 (40)	
Enhancement			
Solid	5 (100)	3 (30)	**0.026**
Heterogeneous or minimal	0 (0)	7 (70)	
Margin			
Well-defined	4 (80)	4 (40)	0.282
Ill-defined	1 (20)	6 (60)	
Hemorrhage/mineralization			
Yes	1 (20)	4 (40)	0.600
No	4 (80)	6 (60)	
Necrosis			
Yes	1 (20)	7 (70)	0.119
No	4 (80)	3 (30)	
Histology			
CMB	5 (100)	7 (70)	0.505
DNMB	0 (0)	3 (30)	

Abbreviations: CMB, classic medulloblastoma; DNMB, desmoplastic/nodular medulloblastoma; ECB, extracerebellar; GTR, gross total resection; ICB, intracerebellar; CN, cranial nerve; STR, subtotal resection.

*Fisher’s exact test (2-sided). ^#^The putative point of origin of the tumor is identified based on the pre- and postoperative MRI results and intraoperative reports.

### *TP53* Mutations

Somatic *TP53* mutations were detected in all SHH-MBs. One of three pediatric MBs (case 14; 33.3%) harbored a missense mutation *of TP53* on exon 8 (NM_000546, c.898 C > A p. P 300 T). In contrast, no *TP53* mutations were observed in adult SHH-MBs.

### Follow-up and prognosis

All patients received adjuvant craniospinal irradiation (30–36 Gy and 54–60 Gy to a primary tumor site) postoperatively, and 14 patients received 4–6 cycles of chemotherapy (Table 1). The follow-up time ranged from 7 months to 135 months, with a mean time of 68.3 months. During follow-up, local recurrence was noted in 5 patients (33.3%), one of whom required emergency surgery because of a cerebellar tonsillar hernia 17 months after the initial surgery.

**Figure 3:**
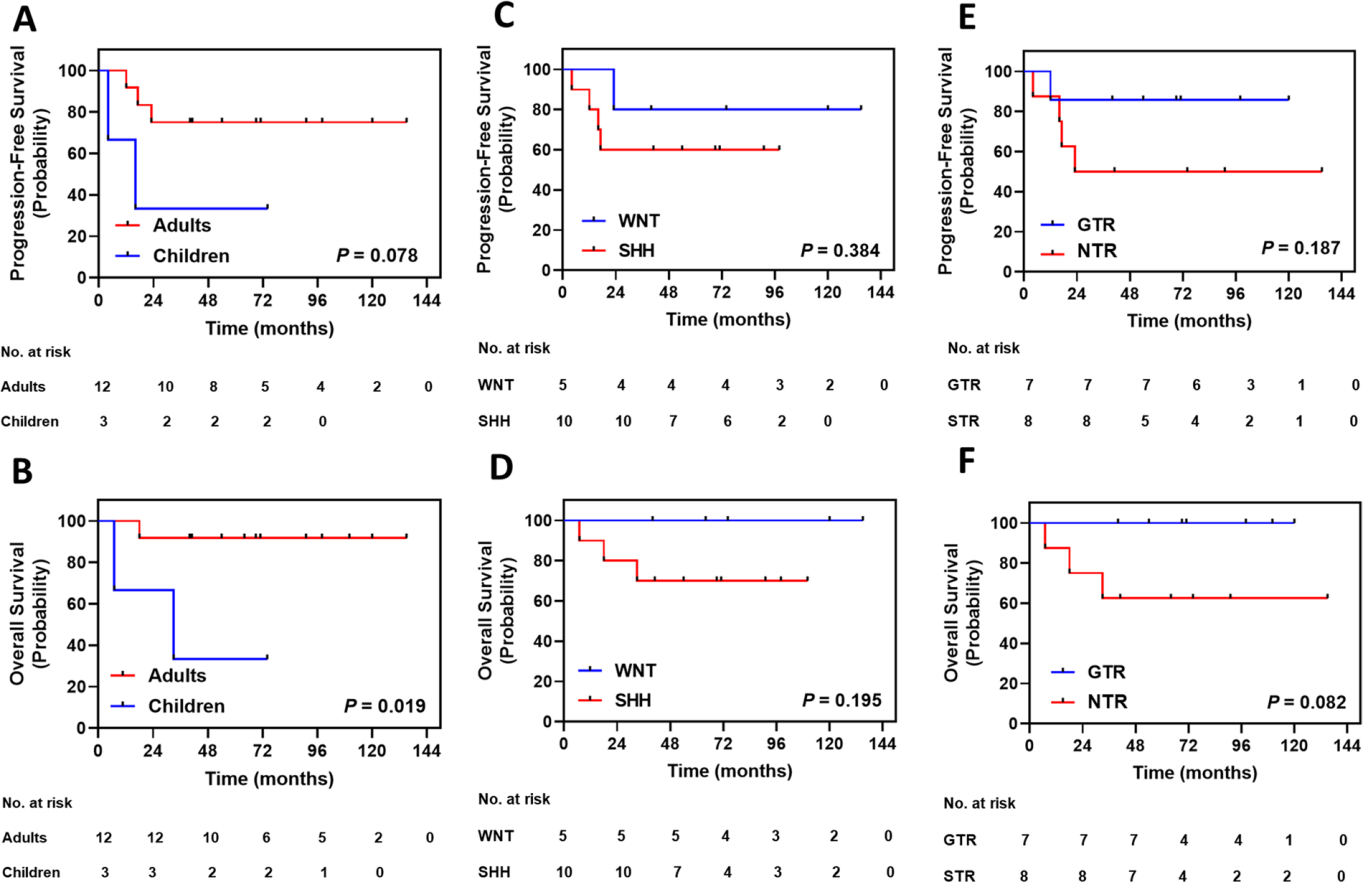
Kaplan-Meier plots of estimated progression-free survival and overall survival time distributions for patients with cerebellopontine angle medulloblastoma (MB; n = 15) according to: (**A**,**B)** Age subgroup — adults versus children; (**C**,**D**) Molecular subgroup — WNT versus SHH; (**E**,**F**) The extent of surgery — gross total resection (GTR) versus near-total resection (NTR); *P* value was determined using the log-rank method.

Three patients died during follow-up (Table 1). The 5-year OS and PFS rates of patients with CPA MB were 80.0% ± 10.3% and 66.7% ± 12.2%, respectively. Kaplan-Meier analysis showed that pediatric patients (5-year OS: 33.3 ± 27.2%) displayed worse OS than adult patients (5-year OS: 91.7 ± 8.0%; *P* = 0.020, Fig. 3B), while the 5-year PFS rate tended to be lower in pediatric patients (5-year PFS: 33.3 ± 27.2%) than in adult patients (5-year PFS: 75.0 ± 12.5%; *P* = 0.078; Fig. 3A). Molecular subgroups conferred no statistical significance upon survival (Fig. 3C, D). The 5-year OS rate tended to be higher in patients with GTR (100%) than in patients with NTR (62.5 ± 17.1%; *P* = 0.082; Fig. 3F), while the 5-year PFS rate was similar between the two groups (Fig. 3E).

## Discussion

MB is one of the most common pediatric intracranial tumors with the preferred site of the cerebellar vermis^23^. A cerebellar hemispheric location is predominantly found in both pediatric and adult SHH-MBs^6^. CPA MB is extremely rare. To date, only 48 cases have been previously described in the current English literature: 21 children and 27 adults with a mean age at diagnosis of 21.3 years (ranging from 0.75–46 years; Table 3)^11–18^. The male to female ratio is 1.1:1. In this series, we analyzed the demographic characteristics, clinical treatments, molecular subgroups and outcomes in a cohort of 15 CPA MBs. To date, this is the largest comprehensive study of this rare tumor. The mean age at diagnosis and the sex ratio in our cohort were similar to those of previous studies (Table 3). Moreover, we found that CPA MB accounted for 5.6% (12/214) of all cases of adult MB and only 0.4% (3/754) of all cases of pediatric MBs. This data confirm the result that CPA MB is more common in adult than in children.

**Table 3:** Literature review of primary cerebellopontine angle medulloblastomas.

Author, year	Case No.	Age	Sex	CNs involved	Resection	Site of origin	Histology	Adjuvant therapy	Status, time* (Mon.)
Xia H, *et al*., 2019	8	7–52 yrs	M = 5; F = 3	Yes	GTR = 6; STR = 2	CE = 7; BS = 1	CMB = 5; DNMB = 3	CT = 3	Alive, 5–34
Satyarthee GD, *et al*., 2018	1	16 yrs	F	Yes	GTR	CE	DNMB	RT	Alive, 6
Noiphithak R, *et al*., 2017	1	2 yrs	F	Yes	GTR	BS	ENMB	CT	Alive, N/A
Bahrami E, *et al*., 2014	1	23 yrs	M	Yes	GTR	N/A	DMB	RT	Alive, N/A
Meshkini, *et al*., 2014	1	7 yrs	F	N/A	N/A	CE	DNMB	N/A	N/A
McLaughlin, *et al*., 2014	1	26 yrs	F	Yes	STR	BS	CMB	RT	N/A
Spina, *et al*., 2013	2	22 yrs; 26 yrs	M = 1; F = 1	Yes	GTR	N/A	CMB	RT	Alive, N/A
Singh, *et al*., 2011	1	22 yrs	M	Yes	GTR	CE	CMB	No	Alive, 15
Furtado, *et al*., 2009	1	32 yrs	M	N/A	GTR	N/A	CMB	N/A	N/A
Yoshimura J, *et al*., 2009	1	23 yrs	F	Yes	STR	BS	CMB	RT + CT	Alive, 15
Fallah, *et al*., 2009	1	47 yrs	M	N/A	STR	CE	CMB	RT	N/A
Nyanaveelan, *et al*., 2007	1	5 yrs	F	Yes	GTR	CE	CMB	RT + CT	Alive, 3–21
Santagata, *et al*., 2007	1	17 yrs	F	Yes	GTR	N/A	MB	RT + CT	N/A
Jaiswal AK, *et al*., 2004	14	3–53 yrs	M = 8; F = 6	Yes	GTR = 7; STR = 7	N/A	CMB = 10; DMB = 4	RT + CT	Alive, 24
Park SY, *et al*., 2004	1	15 yrs	M	Yes	STR	N/A	MB	RT + CT	Alive, 12
Gil-Salu, *et al*., 2004	1	40 yrs	M	Yes	GTR	CE	DMB	RT	N/A
Izycka, *et al*., 2003	1	26 yrs	F	No	N/A	CE	MB	RT	N/A
Akay KM, *et al*., 2003	1	21 yrs	M	N/A	STR	CE	MB	RT + CT	Alive, 18
Kumar R, *et al*., 2001	4	8–24 yrs	M = 3; F = 1	Yes	GTR	N/A	MB = 3; DMB = 1	RT = 1; RT + CT = 3	Alive = 1, 30; Died = 3, N/A
Naim, *et al*., 2000	1	3 yrs	F	Yes	GTR	BS	DMB	RT	Alive, 12
Mehta, *et al*., 1998	1	40 yrs	M	Yes	STR	BS	DMB	RT	Alive, 9
Ahn, *et al*., 1997	1	0.75 yrs	F	Yes	STR	BS	MB	CT	Died, 2
Yamada, *et al*., 1993	1	19 yrs	F	No	STR	CE	MB	RT + CT	N/A
House, *et al*., 1985	1	46 yrs	M	No	STR	N/A	MB	RT	Alive, N/A

Abbreviations: CE, cerebellar hemisphere; CMB, classic medulloblastoma; CT, chemotherapy; DMB, desmoplastic medulloblastoma; DNMB, desmoplastic/nodular medulloblastoma; ENMB, extensive nodularity medulloblastoma; F, female; GTR, gross total resection; M, male; Mon., month; N/A, not applicable; RT, radiotherapy; STR, subtotal resection.

*The time for available follow-up.

The identification of molecular subgroups can provide important opportunities to improve our understanding of the tumor origin and the management of MB in the early postoperative period and at follow-up^23^. Our study identified that CPA MBs consist of two MB molecular subgroups: WNT-MBs in 33% of cases and SHH-MBs in 67% of cases. This finding is consistent with a recent study in which only WNT and SHH subgroups were found in CPA MBs^12^. Moreover, two recent studies demonstrate that the putative cells of origin for WNT- and SHH-MBs seem to be totally different: WNT-MBs arise from the nuclei in the dorsal brainstem, while SHH-MBs arise from the granule neuron precursor cells located in the cerebellar hemispheres^24,25^. In our study, we found that CPA SHH-MBs were characteristic of cerebellar growth without 4^th^ ventricle floor involvement. In contrast, WNT-MBs commonly infiltrated the dorsal and lateral brainstem. These findings indicate that CPA SHH-MBs may arise from the external granular layer of the cerebellar hemisphere, while CPA WNT-MBs may develop from the lateral medullary velum or superficial layers of the dorsolateral brainstem and protrude into the CPA cistern. Moreover, our results further confirm the results of previous studies^2,6,10,26^ showing that WNT- and SHH-MBs have distinct spatial distributions from Group 3- and Group 4-MBs in the posterior fossa.

The prognosis of CPA MB remains uncertain due to the small number and short-term follow-up of reported cases. In our cohort, there was no significant difference in OS or PFS between CPA WNT- and SHH-MBs. The possible reason of the similar outcome between two subgroups is that most CPA MBs occur predominantly in adult patients, in which WNT-MBs demonstrate a similar outcome as SHH-MBs^21,27^. Interestingly, we observed that age may be a potential prognostic factor in CPA MB. We found that 67% of pediatric patients died during the follow-up, while the death rate was only 8.3% in adult group. This finding is coincident with previous data, in which 75% of deaths are children (as shown in Table 3). Moreover, we observed that adults with CPA MB demonstrated a good prognosis, the 5-year OS (91.7%) and PFS (75.0%) rates were even higher than those of other adult MBs (OS: 73%; PFS: 60%). In total, our and previous results indicate that pediatric patients with CPA MB may need additional clinical care.

It is challenging to achieve GTR due to the tumor involvement of CNs, the cerebellum and brainstem, though we observed that the OS for CPA MB tended to benefit from GTR. As shown in Table 3, GTR are performed only in approximately 50% of all cases, and the majority of these tumors have involved CNs, including the fifth, seventh, and eighth cranial nerves. Although maximal safe removal remains the standard of care to prolong the period of survival for patients with MB, aggressive resection for CPA MB may increase the surgical complications. We like to emphasize that all patients in our study are benefited from generous tumor debulking and decompression of the brainstem. And surgical removal of small residual portions of tumors adherent to CNs or the brainstem should not be recommended when the likelihood of neurological morbidity is high.

The main limitation of this study is its retrospective nature and relatively small patient population, which undoubtedly limited the statistical power of some of our analyses. Future retrospective multicenter studies with larger data sets will be required to validate our findings.

## Conclusions

In conclusion, our study demonstrates that MB located in the CPA is extremely rare and commonly occurs in adults. Two molecular subgroups are observed in this rare tumor. Adult patients with CPA MB usually have a good prognosis, while a poor outcome is observed in pediatric patients. Our findings could be helpful for developing a subsequent management plan when treating this rare disease.

## Supplementary information

Dataset 1.
