# Supplementary material for: The clinical treatment and outcome of cerebellopontine angle medulloblastoma: a retrospective study of 15 cases
Source: Sci Rep. 2020 Jun 17;10:9769. doi: 10.1038/s41598-020-66585-7 (PMC7300107; doi:10.1038/s41598-020-66585-7)
Supplement: Supplementary file 1 — Dataset 1. [file 41598_2020_66585_MOESM1_ESM.pdf]

The clinical treatment and outcome of cerebellopontine angle medulloblastoma: a retrospective study of 15 cases

Tao Wu[1,4], Pei-ran Qu[1,3,4], Shun Zhang[2], Shi-wei Li[1], Jing Zhang[1], Bo Wang[2], Pinan Liu[1,2], Chun-de Li[1,2], and Fu Zhao[1,2]

Supplementary table 1. The comparison of clinical characteristics between adult and pediatric cerebellopontine angle medulloblastomas

Characteristics	Children (n=3) No. of patients (%)	Adults (n=12) No. of patients (%)	*P* *
Gender			
Female	3 (100)	4 (33)	0.077
Male	0 (0)	8 (67)	
Ataxia			
Yes	2 (67)	12 (100)	0.200
No	1 (33)	0 (0)	
Diplopia			
Yes	0 (0)	4 (33)	0.516
No	3 (100)	8 (67)	
Hearing loss			
Yes	0 (0)	4 (33)	0.516
No	3 (100)	8 (67)	
Cystic			
Yes	3 (100)	6 (50)	0.229
No	0 (0)	6 (50)	
Hemorrhage/ mineralization			
Yes	2 (67)	3 (25)	0.242

No	1 (33)	9 (75)	
Necrosis			
Yes	2 (67)	6 (50)	1.000
No	1 (33)	6 (50)	
Enhancement			
Solid	1 (33)	7 (58)	0.569
Heterogeneous or minimal	2 (67)	5 (42)	
Margin			
Well-margin	1 (33)	7 (58)	0.569
Ill-margin	2 (67)	5 (42)	
Histology			
Classic MB	3 (100)	9 (75)	1.000
DNMB	0 (0)	3 (25)	
Molecular subgroup			
WNT	1 (33)	4 (33)	1.000
SHH	2 (67)	8 (67)	

Abbreviations: PCN, posterior cranial nerves; DN, desmoplastic nodular.

* Fisher's exact test.
